# Between-day reliability of centre of pressure measures for balance assessment in hemiplegic stroke patients

**DOI:** 10.1186/1743-0003-11-39

**Published:** 2014-03-21

**Authors:** David Gasq, Marc Labrunée, David Amarantini, Philippe Dupui, Richard Montoya, Philippe Marque

**Affiliations:** 1 Explorations Fonctionnelles Physiologiques, CHU Toulouse Rangueil, Avenue du Pr Jean Poulhès, 31059 Toulouse, France; 2 PRISSMH, Université Paul Sabatier Toulouse 3, route de Narbonne, 31062 Toulouse, France; 3 Inserm, Institut des maladies métaboliques et cardiovasculaires, UMR 1048, Equipe 8, F-31432 Toulouse, France; 4 Unité de Rééducation Cardiovasculaire, CHU Toulouse Rangueil, Avenue du Pr Jean Poulhès, 31059 Toulouse, France; 5 Inserm, Imagerie cérébrale et handicaps neurologiques, UMR 825, F-31059 Toulouse Cedex 9, France; 6 Université de Toulouse, UPS, Imagerie cérébrale et handicaps neurologiques, UMR 825, CHU Purpan, Place du Dr Baylac, F-31059 Toulouse Cedex 9, France; 7 Département de kinésiologie, Université de Montréal, boulevard Édouard-Montpetit, Montréal, Québec; 8 Service de Médecine Physique et Réadaptation, CHU Toulouse Rangueil, Avenue du Pr Jean Poulhès, 31059 Toulouse, France

**Keywords:** Stroke, Postural balance, Reproducibility of results, Bias, Heteroscedasticity, Posturography, Centre of Pressure (CoP)

## Abstract

**Background:**

Stroke patients have impaired postural balance that increases the risk of falls and impairs their mobility. Assessment of postural balance is commonly carried out by recording centre of pressure (CoP) displacements, but the lack of data concerning reliability of these measures compromises their interpretation. The purpose of this study was to investigate the between-day reliability of six CoP-based variables, in order to provide i) reliability data for monitoring postural sway and weight-bearing asymmetry of stroke patients in clinical practice and ii) consistent assessment method of measurement error for applications in physical medicine and rehabilitation.

**Methods:**

Postural balance of 20 stroke patients was assessed in quiet standing on a force platform, in two sessions, 7 days apart. Six CoP-based variables were collected in eyes open and eyes closed conditions: postural sway was assessed with mean and standart deviation of CoP-velocity, CoP-velocity along the mediolateral and anteroposterior axes, and confidence ellipse area (CE_AREA_); weight-bearing asymmetry was assessed with mean CoP position along the mediolateral axis (CoP_ML_). The intraclass correlation coefficient (ICC) was used to determine the level of agreement between test-retest. Small real difference (SRD), corresponding to the smallest change that indicates a real improvement for a single individual, was used to determine the extent of measurement error.

**Results:**

ICCs were satisfactory (>0.9) for all CoP-based variables, except for CE_AREA_ in eyes open condition and CoP_ML_ (<0.8). The SRDs (eyes open/closed conditions) were: 6.1/9.5 mm.s^-1^ for mean velocity; 12.3/12.2 mm.s^-1^ for standard deviation of CoP-velocity; 3.6/5.5 mm.s^-1^ and 4.9/7.3 mm.s^-1^ for CoP-velocity in mediolateral and anteroposterior axes, respectively; 17.4/21.4 mm for CoP_ML_. Because CE_AREA_ showed heteroscedasticity of measurement error distribution, SRD (eyes open/closed conditions) was expressed as a percentage (121/75%) and a ratio (3.68/2.16) obtained after log-antilog procedure.

**Conclusions:**

In clinical practice, the CoP-based velocity variables should be prefer to CE_AREA_ to assess and monitor postural sway over time in hemiplegic stroke patients. The poor reliability of CoP_ML_ compromises its use to assess weight-bearing asymmetry. The procedure we used could be applied in reliability studies concerning other CoP-based variables or other biological variables in the field of physical medicine and rehabilitation.

## Background

Balance disorders are common following a stroke, with consequences in terms of increased risk of falling, marked limitations in activities of daily living and for walking, and risk of death [1]–[5]. Compared to healthy subjects, stroke patients have postural balance impairments that result in increased postural sway and weight-bearing asymmetry in quiet standing, that is commonly carried out by recording centre of pressure (CoP) displacements with a force platform [6]–[11]. CoP-based findings are directly related to clinical impairment of balance and gait [3,5,12-17], and have important implications for clinical practice to monitor postural recovery [8], assess the risk of falls [7], evaluate the effectiveness of rehabilitation programs [18] or in addition to clinical functional tests to measure different aspects of balance control [17,19]. Accurate assessment of CoP measures in hemiplegic stroke patients is of particular interest to clinicians when clinical balance scales, such as the Postural Assessment Scale for Stroke Patients [20], show a ceiling effect.

Like many biological measurements, CoP measures have an intrinsic variability that affects their test-retest reliability as well as the validity and responsiveness of postural control assessment. Identifying measurement error of CoP measures in patients is fundamental for clinicians, to ensure that any observed modification in CoP measures between two sessions reflects real change in postural control capacities, rather than random or systematic error in the measurement procedure [21-23]. Intraclass correlation coefficient (ICC) has become a popular statistical choice in reliability studies to assess the agreement between measurements on two sessions. However, a more comprehensive evaluation of reliability, suitable for monitoring changes in the performance of a subject over time in clinical practice, should include assessment of the extent of measurement error with, for instance, standard error of measurement (SEM) and small real difference (SRD) that are directly applicable to recorded data [21,22,24]. Moreover, reliability is not a fixed property and depends on the population studied [25]. To date, many reliability studies have focused on healthy subjects [25-30], or patients with different levels of disequilibrium [31,32], but no study has specifically and comprehensively investigated the test-retest reliability of CoP-based variables in quiet standing for hemiplegic stroke population.

The aim of this study was to investigate the between-day reliability of six CoP-based variables that have relevance in hemiplegic stroke patients, in order to provide i) reliability data for monitoring postural sway and weight-bearing asymmetry of stroke patients in clinical practice and ii) consistent assessment method of measurement error for applications in physical medicine and rehabilitation.

## Methods

### Participants

Twenty subjects with hemiparesis due to a single cerebrovascular accident (14 males and 6 females, 11 left and 9 right hemiparesis, mean age: 49.7 ± 15 years, mean time since stroke: 10.3 {from 1 to 37} months, mean Fugl-Meyer scale for lower limb [33]: 23 ± 7.9 /34, mean Postural Assessment Scale for Stroke Patients [20]: 33.2 ± 3 /36, mean Functional Independence Measure [34]: 107.4 ± 13.2 /126) were recruited. An additional file shows the detailed clinical characteristics for each patient [see Additional file 1]. No differences were found for clinical characteristics between male and female or between right and left hemiplegics (p > 0.05).

Ethics approval was obtained from the local ethics committee of the Paul Sabatier University Hospital, Toulouse, France (*Comité d’éthique de la recherche du CHU de Toulouse*). All participants signed an informed consent form (including the agreement for publication of anonymized data) according to the Declaration of Helsinki recommendations for investigations with human participants.

The inclusion criteria corresponded to patients at least one month post-stroke, able to stand independently for at least five minutes without assistance. Exclusion criteria included subjects with musculoskeletal or neurological disorders in addition to stroke, or subjects with concomitant cognitive or psychiatric problems that impaired their ability to follow simple verbal instructions. Patient recruitment was performed during follow-up visits in a neurological rehabilitation unit. During the visit, patients were included if they presented no exclusion criteria and gave their consent to participate in the study. The inclusions were conducted from April 2008 to June 2009.

### Experimental design

The study was designed as a test-retest reliability study, with a 7-day interval between two sessions, carried out at the same time of day, without modifying medication. In each session, subjects were submitted to CoP measures under two alternating visual conditions: eyes open and eyes closed. In eyes open condition, subjects were asked to look straight ahead at a fixed target 2 m away. In both eyes conditions, their feet were placed barefoot in a standardized position, heels 3 cm apart and toes pointed out at an angle of 30°. The subjects were instructed to sway as little as possible (quiet standing) for 3 trials in each visual condition. Each trial lasted 51.2 seconds, with a seated rest of 1 min between each.

### Data recording and processing

A force platform (Win-Posturo, Medicapteurs, Toulouse, France; CE Dekra certification directive 93/42 appendix VI, 16 bits A/D conversion) with three strain gauges (sensitivity: 0.01 N; hysteresis: 0.2%; linearity: 0.2%; active lowpass filter: 106 Hz) measuring the vertical ground reaction force at 40 Hz, was used to obtain a two dimensional analysis of CoP displacements along both the anteroposterior and mediolateral axes of the platform. The size of the rigid plate constituting the upper part of the platform was 460 mm × 460 mm. Transducers initialization was performed prior to each series of recordings. Data were saved and processed via the WinPosture NV 1.6™ software package.

Although many CoP-based variables have been proposed in the literature, we focused on six of them that have already been studied and demonstrated their relevance in hemiplegic stroke patients [8,10-15,17,19,35]:mean and standard deviation of resultant CoP velocity (VEL and SD_VEL_, respectively, in mm.s^-1^), mean velocity of CoP along the mediolateral and anteroposterior axes (VEL_ML_ and VEL_AP_, respectively, in mm.s^-1^), area of the 90% confidence ellipse enclosing CoP (CE_AREA_ in mm^2^), and absolute value of the mean CoP position along the mediolateral axis (CoP_ML_ in mm). The weight-bearing asymmetry was assessed with CoP_ML_, and postural sway with the other five CoP-based variables. For each variable, the mean of the 3 trials obtained from each participant in eyes open and eyes closed conditions was used for data analysis.

### Data analysis

Normal distribution of the data was verified via the Kolmogorov-Smirnov test. Absence of significant systematic bias was inferred when zero was included in the 95% confidence interval for the mean of the individual test-retest differences [24,36].

Each variable was then analysed in two steps.

The first step was analysis of random error (i.e., individual test-retest differences) distribution led to evaluation of the homoscedasticity/heteroscedasticity of the data. Heteroscedasticity refers to proportionality between random error and the individual mean of the two sessions (i.e., a larger random error is associated with a larger measurement) [21,22,36,37]. A previously reported method was employed to address potential heteroscedasticity in the data: calculation of Pearson’s *r* correlation coefficient between absolute individual test-retest differences and individual means of the two sessions. A positive and significant *r* was interpreted as evidence of heteroscedasticity in the data [21,36]. Graphical illustration (Bland-Altman plot [36]), which charts random error against the individual mean of the two sessions, was used to visualize the direction of the dispersion around the zero line. The distribution was considered heteroscedastic when a larger random error is associated with a larger measurement.

The second step was to estimate the reliability of the data using some parameters from the literature. For all CoP-based variables, ICC were used to determine the test-retest level of agreement regardless of the distribution of the random error. ICC_2,k_ for mean of k measures (k = 3) were used to account for a random effect over time [38]. We consider as satisfactory for individual comparisons an ICC greater than 0.9 [39].

The extent of measurement error was estimated using SEM and SRD. In this framework, the calculation procedures differed according to the presence or absence of heteroscedasticity of random error. In the absence of heteroscedasticity, SEM and SRD were expressed in the original units of measurement. SEM was obtained using the square root of the within-subject error variance [24], which is equivalent to the typical error proposed by Hopkins [22]. SEM corresponds to the 68th percentile of measurement error, and represents the smallest change that indicates a real improvement for a group of individuals [21,24]. SEM was calculated with:

$$\mathrm{SEM}=\mathrm{SDdiff}/\sqrt{2}$$

where SDdiff is the standard deviation of individual test-retest differences.

SRD, introduced by Beckerman et al. [23], is algebraically similar to the limit of agreement previously described [22,36]. SRD corresponds to the 95th percentile of measurement error, and represents the smallest change that indicates a real improvement for a single individual [21,24]. SRD was calculated with:

$$\mathrm{SRD}=t_{0.975,f}\times \mathrm{SEM}\times \sqrt{2}=t_{0.975,f}\times \mathrm{SDdiff}$$

where t_0.975,f_ is the value of the t statistic with a cumulative probability of 0.975 and *df* degrees of freedom (*df*  = 19 and t_0.975,f_  = 2.093).

In the presence of heteroscedasticity, two different methods were used to determine the extent of measurement error as suggested by some authors [21,22]:

- Natural logarithmic transformation was performed on measurement error according to previous publications [21,36,37]. Natural logarithmic transformation yielded a ratio ≥ 1 (a value of 1 corresponds to maximum reliability) for SEM and SRD (specifically, SEM_R_ and SRD_R_). SEM_R_ and SRD_R_ were calculated with:

$${\mathrm{SEM}}_{R}={\mathrm{aSDdiff}}^{\left(1/\surd 2\right)}$$

$${\mathrm{SRD}}_{R}={\mathrm{aSDdiff}}^{\left(t0.975,f\right)}$$

where aSDdiff is the antilog of SDdiff.

- SEM and SRD were expressed as percentages (SEM_P_ and SRD_P_) independently of the original units. The proposed method is not a simple division of the measurement error in absolute value (i.e. SEM or SRD) by the mean of all the measurements from both sessions. The within-subject variability has been taking into account using the standard deviation of the individual test-retest differences expressed as a percentage of the individual means of the two sessions. SEM_P_ and SRD_P_ were calculated with:

$${\mathrm{SEM}}_{p}={\mathrm{SDdiff}}_{p}/\sqrt{2}$$

$${\mathrm{SRD}}_{p}=t_{0.975,f}\times {\mathrm{SEM}}_{p}\times \sqrt{2}=t_{0.975,f}\times {\mathrm{SDdiff}}_{p}$$

where SDdiff_P_ is the standard deviation of the individual test-retest differences (SDdiff) expressed as a percentage of the individual means of the two sessions.

## Results

### Distribution of random error

**Figure 1:**
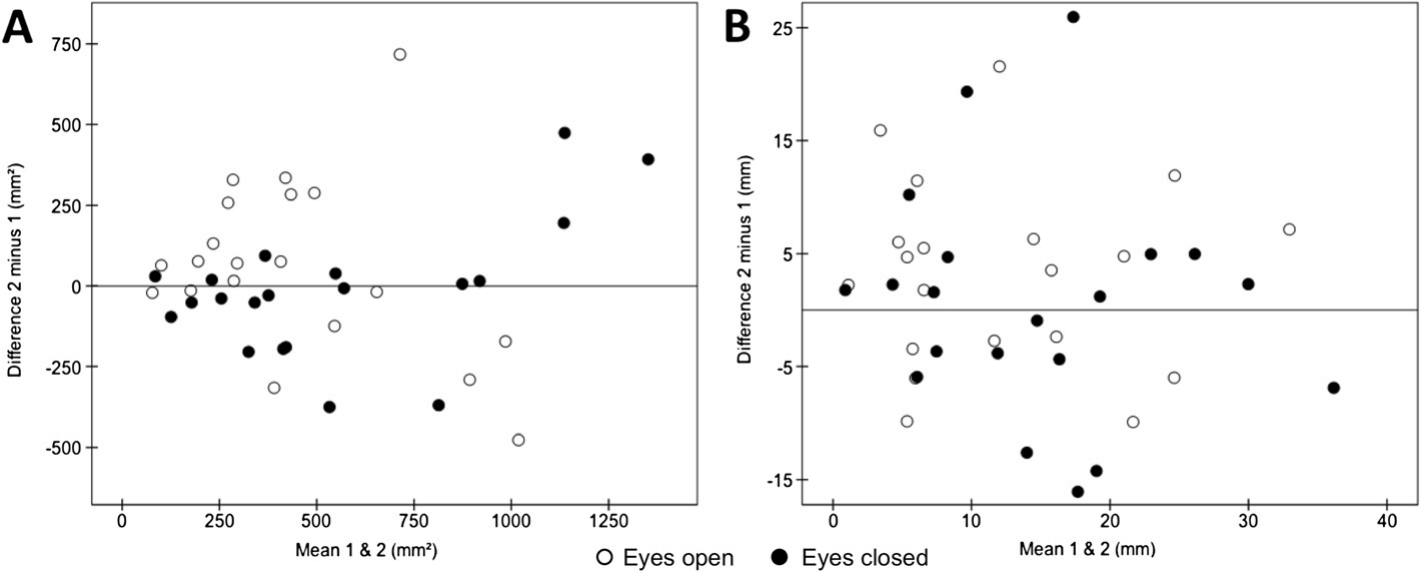
**Graphic illustrations of random error distribution for CE**_**AREA **_**and CoP**_**ML.**_ Random error (i.e. individual test-retest differences) is plotted against the individual means of the two sessions, both in eyes open and eyes closed conditions. For CE_AREA_**(A)** the larger random error for higher mean values is suggestive of heteroscedasticity. For CoP_ML_**(B)** the random error is rather constant whatever the mean, which suggests absence of heteroscedasticity.

Zero was included in the 95% confidence interval for the mean of the individual test-retest differences for all CoP-based variables, which excludes a learning effect between the two sessions (Table 1). Pearson’s *r* correlation coefficient between the absolute random error and the individual mean of the two test sessions was significant only for CE_AREA_ in both eyes open condition (*r* = 0.52, p = 0.02) and eyes closed condition (*r* = 0.56, p = 0.01), which indicated heteroscedasticity of random error distribution (Table 1). Visual interpretation of random error distribution also indicated heteroscedasticity for CE_AREA_ in both eyes open and eyes closed conditions, as a larger random error is associated with a larger measurement (Figure 1A). For the five other variables, there was no argument for heteroscedasticity of random error distribution (Table 1 for insignificant Pearson’s *r* correlation coefficients; graphical illustration for CoP_ML_ in Figure 1B and in additional file for other variables [see Additional file 2]).

**Table 1:** Descriptive data of CoP-based variables and characterization of the random error distribution

**CoP-based variables**	**EO/EC**	**Session 1**	**Session 2**	***d *** **(95% CI)**	***r***	**P-values**
		**Mean ± SD**	**Mean ± SD**			
VEL (mm.s^-1^)	EO	14.8 ± 5.2	15.0 ± 6.7	0.1 (-1.2 to 1.5)	0.15	0.54
	EC	21.3 ± 11.3	21.4 ± 13.4	0.1 (-2.0 to 2.2)	0.34	0.15
SD_VEL_ (mm.s^-1^)	EO	37.7 ± 12.3	37.8 ± 13.5	0.1 (-2.6 to 2.9)	0.10	0.66
	EC	38.1 ± 11.6	38.1 ± 12.8	0.0 (-2.7 to 2.7)	0.25	0.29
VEL_ML_ (mm.s^-1^)	EO	7.6 ± 3.1	7.6 ± 4.0	0.0 (-0.8 to 0.8)	0.27	0.26
	EC	10.7 ± 5.8	10.6 ± 7.0	-0.1 (-1.3 to 1.1)	0.32	0.17
VEL_AP_ (mm.s^-1^)	EO	10.1 ± 3.7	10.3 ± 4.7	0.3 (-0.8 to 1.4)	0.21	0.38
	EC	15.1 ± 8.6	14.8 ± 8.4	-0.3 (-2.0 to 1.3)	0.40	0.08
CE_AREA_ (mm^2^)	EO	438.3 ± 229.2	449.5 ± 377.8	11.2 (-188.6 to 140.9)	0.52	0.02*
	EC	558.6 ± 324.0	541.2 ± 431.5	-17.4 (-115.5 to 80.7)	0.56	0.01*
CoP_ML_ (mm)	EO	10.7 ± 9.9	13.8 ± 9.6	3.1 (-0.8 to 7.0)	0.05	0.82
	EC	14.1 ± 11.6	14.7 ± 9.9	0.5 (-4.3 to 5.3)	0.03	0.92

For each of the six CoP-based variables, mean ± standard deviation in session 1 and 2, and mean difference between the 2 test sessions (*d*) with its 95% confidence interval (95% CI) are reported in eyes open (EO) and eyes closed (EC) conditions. The detection of a possible heteroscedasticity of the random error distribution was done with calculation of Pearson’s correlation coefficient (*r*) between absolute individual test-retest differences and individual means of the two sessions. Significant p-values (p < 0.05) are indicated with an asterisk.

### Determination of the level of agreement

The reliability data of CoP-based variables are presented in Table 2. ICC values for CE_AREA_ in eyes open condition and for CoP_ML_ in both eyes open and eyes closed conditions were not satisfactory, respectively 0.76, 0.78 and 0.71. For all the other variables, ICC values were greater than 0.90 and the lower 95% confidence interval values were greater than 0.75. VEL in eyes closed condition showed the higher ICC value, 0.97. Level of agreement was quite similar in eyes open and closed conditions.

**Table 2:** Reliability data of CoP-based variables

**CoP-based variables**	**EO/EC**	**ICC**_**2,k **_**(95% CI)**	**SD/SD**_**P **_**(o.u /%)**	**SEM/SRD (o.u.)**	**SEM**_**P**_**/SRD**_**P **_**(%)**	**SEM**_**R**_**/SRD**_**R **_**(ratio)**	***SRD***_***MP ***_ ***(%)***
VEL (mm.s^-1^)	EO	0.94 (0.84-0.98)	2.9/…	2.1/6.1	…	…	*41*
	EC	0.97 (0.91-0.99)	4.5/…	3.2/9.5	…	…	*45*
SD_VEL_ (mm.s^-1^)	EO	0.95 (0.86-0.98)	5.9/…	4.1/12.3	…	…	*33*
	EC	0.94 (0.85-0.98)	5.8/…	4.1/12.2	…	…	*32*
VEL_ML_ (mm.s^-1^)	EO	0.94 (0.84-0.98)	1.7/…	1.2/3.6	…	…	*47*
	EC	0.96 (0.89-0.98)	2.6/…	1.9/5.5	…	…	*51*
VEL_AP_ (mm.s^-1^)	EO	0.92 (0.79-0.97)	2.3/…	1.6/4.9	…	…	*48*
	EC	0.96 (0.89-0.98)	3.5/…	2.5/7.3	…	…	*51*
CE_AREA_ (mm^2^)	EO	0.76 (0.38-0.90)	…/57.9	…	41/121	1.55/3.68	*131*
	EC	0.92 (0.79-0.97)	…/35.7	…	25/75	1.30/2.16	*80*
CoP_ML_ (mm)	EO	0.78 (0.44-0.91)	8.3/…	5.9/17.4	…	…	*141*
	EC	0.71 (0.27-0.88)	10.2/…	7.2/21.4	…	…	*149*

For each of the six CoP-based variables, reliability data were reported in eyes open (EO) and eyes closed (EC) conditions. For all the CoP-based variables, ICC_2,k_ with its 95% confidence interval (95% CI) and SRD expressed in percentage of the mean measurement between session 1 and 2 (*SRD*_*MP*_, in the last column, not to use for clinical practice but to compare the magnitude of the measurement error between the different CoP-based variables) were reported. For CoP-based variables without heteroscedastic distribution of random error distribution, standard deviation of random error (SD), standard error of measurement (SEM) and small real difference (SRD) expressed in the original units of measurement (o.u.) were reported. For CE_AREA_, with heteroscedastic distribution of random error distribution, standard deviation of random error expressed in percentage of the individual means (SD_P_), SEM and SRD expressed in percentage (SEM_P_ and SRD_P_ in%) or as a ratio (SEM_R_ and SRD_R_, obtained after natural logarithmic transformation) were reported.

### Determination of the extent of measurement error

**Figure 2:**
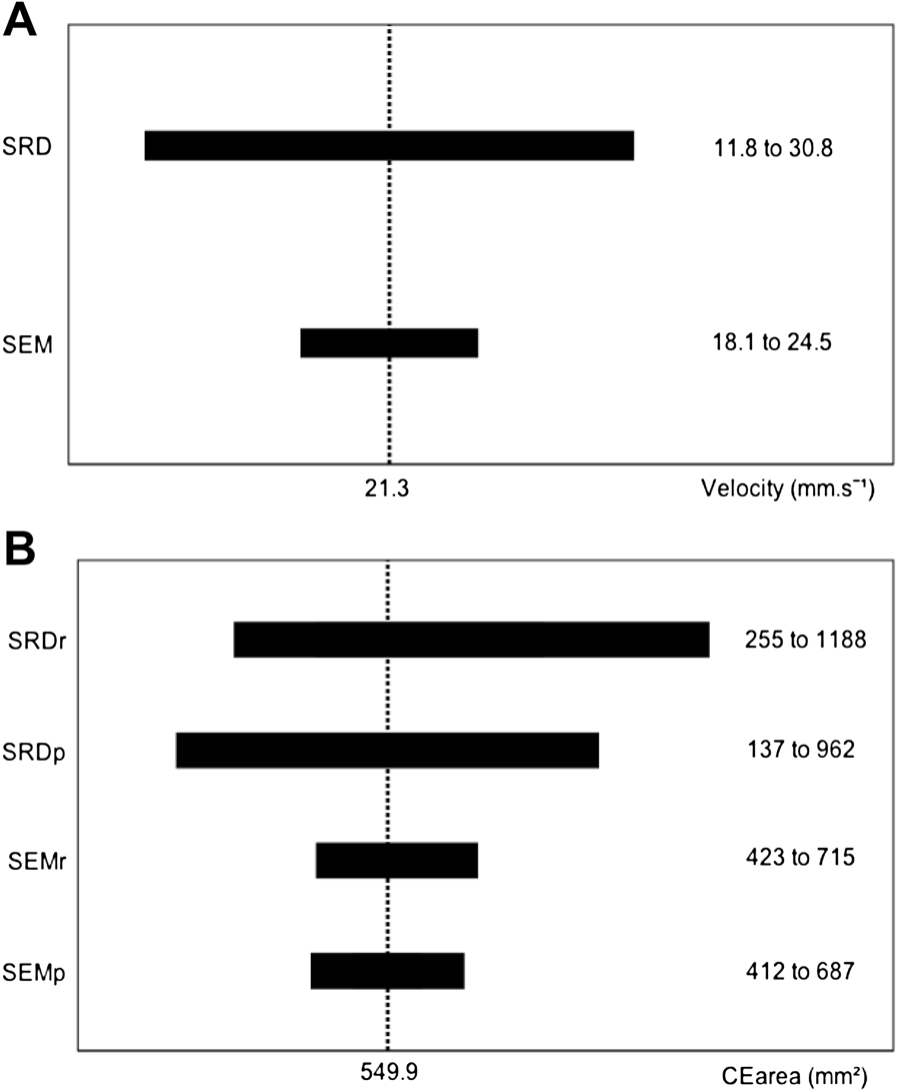
**Extent of the measurement error around the mean value of VEL and CE**_**AREA.**_ The figure shows the extent of measurement error around the average value of VEL and CE_AREA_ obtained in our population, in eyes closed condition. Extent of measurement error was expressed with SRD and SEM for VEL because of absence of heteroscedacticity **(A)**, and with SRD_R_, SRD_P_, SEM_R_ and SEM_P_ for CE_AREA_ because of heteroscedasticity of random error **(B)**.

With regard to the proposed analysis procedure, for VEL, SD_VEL_, VEL_ML_, VEL_AP_ and CoP_ML_ (absence of heteroscedasticity in both eyes open and eyes closed conditions), the extent of measurement error was expressed in the original units of measurement with SEM and SRD, directly usable in clinical practice (Table 2). E.g., for a given stroke patient, use of SRD indicates that a minimal change of 9.5 mm.s^-1^ is necessary to confirm a modification of VEL in eyes closed condition, regardless of its initial value. Figure 2A illustrates the extent of the measurement error obtained by applying SRD and SEM to the average value of VEL in our population (21.3 mm.s^-1^ in eyes closed condition).

In contrast, for CE_AREA_, distribution of the random error showed heteroscedasticity in both eyes open and eyes closed conditions. Accordingly, the extent of measurement error was expressed as a percentage (with SEM_P_ and SRD_P_) and a ratio (with SEM_R_ and SRD_R_) (Table 2). E.g., using CE_AREA_ in eyes closed condition, a minimal change of 75% of the initial value (when using SRD_P_) or a minimal change below *initial value* ÷ *2.16* or above *initial value 2.16* (when using SRD_R_) is required to confirm a modification of postural sway for a given stroke patient. Figure 2B illustrates the extent of the measurement error obtained by applying SRD_R_, SRD_P_, SEM_R_ and SEM_P_ to the average value of CE_AREA_ in our population (549.9 mm^2^ in eyes closed condition). Due to the method of calculation [21], an asymmetric range of measurement error around the mean was found for SRD_R_ and SEM_R_, unlike for SRD_P_ and SEM_P_, and the extent of measurement error around the mean was slightly higher with the log-antilog procedure. E.g., based on the average value of CE_AREA_ in our population (eyes closed condition), range of SRD_P_ was 825 mm^2^ (962 minus 137) versus 933 mm^2^ (1188 minus 255) for SRD_R_ (Figure 2B).

## Discussion

This study provides reliability data and proposes consistent assessment of measurement error for interpretation of changes in postural sway or in weight-bearing asymmetry between two postural control assessments in hemiplegic stroke patients. For each of six relevant CoP-based variables, our approach was to determine the level of agreement using ICC, then to determine the extent of measurement error using SEM and SRD, taking into account random error distribution. In the presence of heteroscedasticity of random error distribution, we determined the extent of measurement error using ratio (with SEM_R_ and SRD_R_) and tested the use of measurement error expressed as percentages (with SEM_P_ and SRD_P_).

### Level of agreement with ICC

ICC values were not satisfactory (i.e., lower than 0.9) for CE_AREA_ in eyes open condition and for CoP_ML_ in both eyes open and eyes closed conditions, whereas ICC values were satisfactory (i.e., ICC greater than 0.9) for other CoP-based variables [39]. The values of ICC obtained in the present study were higher than those reported in Marigold and Eng [10] (0.63 and 0.90 for root-mean-square of CoP displacement and velocity, respectively), which may be due to the longer duration of signals acquisitions (51.2 vs. 30 seconds) and to averaging data obtained from 3 (vs. 2) acquisitions [28]. In stroke patients, CE_AREA_-based variable has already shown moderate reliability (ICC = 0.63) [40]. Previous studies reporting lower reliability for CE_AREA_ in non-stroke subjects corroborate this finding [26,29,30,32]. These data suggest that VEL, SD_VEL_, VEL_ML_, VEL_AP_ are more reliable than CE_AREA_-based variable to assess postural sway in hemiplegic stroke patients. Moderate reliability of CoP_ML_ may be related to the fact that this variable includes two parameters in its calculation: weight distribution and point of application of the reaction force under each foot [41]. Accordingly, the use of two platforms (one under each foot) may be useful for assessing directly the weight-bearing asymmetry under each foot, regardless of the point of application of the reaction force [8,10].

Our data showed firstly an increase of postural sway when vision was removed, which is in accord with the strong reliance on visual information reported in stroke patients [42]. Secondly, for the more reliable CoP-based variables (VEL, VEL_ML_, VEL_AP_, and SD_VEL_), values of ICC were quite similar in eyes open and closed conditions. The removal of vision necessarily increases the reliance on vestibular and somatosensory information, and is accompanied by a strategy that is at least as consistent as eyes open condition to maintain balance [40].

Whereas the ICC has become a popular statistical choice in reliability studies, it is now broadly accepted that using only ICC for a reliability study can lead to erroneous conclusions. Indeed, even a high value does not mean that reliability is acceptable in clinical practice. ICC assesses agreement between repeated measurements and thereby only the variance between subjects, so it is affected by sample heterogeneity [21,22,24]. Finally, in clinical practice it is useful to know the minimal significant change between two assessments, based on the determination of the extent of measurement error in original units or in percentage, with SEM and SRD for instance, that are directly applicable to recorded data [21,22,24].

### Problematic of the random error distribution

Using the determination of measurement error in the original unit (e.g., SEM or SRD) without underscoring heteroscedasticity is problematic because subjects with larger random errors have a greater influence on the calculation of measurement error. Accordingly, measurement error of small values is overestimated and that of the large values is underestimated. In the absence of heteroscedasticity, the inverse problem is encountered when using a parameter expressed as a percentage, such the coefficient of variation or the SRD expressed in percentage of the mean of all the measurements from both sessions for example [22,36]. Heteroscedasticity of random error is common when assessing the measurement error of variables recorded on a ratio scale in sports medicine [43]. The methods used in the present study clearly indicated heteroscedasticity for one of the six variables: CE_AREA_. Together with the presence of heteroscedastic distribution of random error is confirmed for CE_AREA_, it could be concluded that using measurement error parameters expressed in absolute value is inappropriate for this variable, and thus not to be recommended.

Heteroscedasticity might frequently be encountered in the study of biological variability of human performance [21,22,43] such as postural control, but it has not been studied [26-32,44,45], although appropriately taking into account random error distribution improves the relevance of the results.

### Determination of the measurement error for clinical practice

This study proposes reliability data for monitoring postural sway of stroke patients in clinical practice. SEM and SRD, which are proportional to each other, represent the smallest change that indicates a real improvement (i.e. the minimal significant change of score) for a cohort and an individual patient, respectively [21,24]. Generally, the lower the value of the SEM or SRD, the better the reliability of the measurement.

To our knowledge, the only available measurement error data on postural sway from stroke patients reported SEM values ranging from 1.3 to 4.6 mm.s^-1^ for the root-mean-square CoP velocity measures [10]. These values are very close to the SD_VEL_ values of the SEM provided in eyes open and eyes closed conditions in the current study (4.1 mm.s^-1^ in both conditions). The extent of measurement error of CoP-based variables seem to be lower in healthy subjects [29] (for example, SRD of VEL_AP_ in eyes closed condition was 3.3 mm.s^-1^ vs. 7.3 mm.s^-1^ in our study) or populations with moderate musculoskeletal disorders [32] (for example, SRD of VEL in eyes closed condition was 3.1 mm.s^-1^ vs. 9.5 mm.s^-1^ in our study), probably because they have no major balance disorders, unlike the hemiplegics. This illustrates that the data reliability must be determined for each patient population. A priori, for stroke patients, the reliability data obtained in healthy subjects are not usable because the measurement error is much higher.

Knowledge of the measurement error allows the interpretation of the change in postural balance of a stroke patient before and after a rehabilitative program. For VEL, based on present study where ICC showed the better level of agreement, use of SRD indicates that a minimal change of 6.1 mm.s^-1^ (eyes open condition) or 9.5 mm.s^-1^ (eyes closed condition) is necessary to confirm a modification of postural sway, regardless of the initial value. For example, for a given stroke patient assessed before and after a rehabilitative program aimed to improve the balance, if VEL (in eyes open condition) has decreased by 2 mm.s^-1^, it is likely that this change is related to a measurement error. Conversely, if VEL (in eyes open condition) has decreased of 10 mm.s^-1^, it is likely that the patient has really improved his balance. For CoP_ML_, where ICC showed moderate reliability, use of SRD indicates that a minimal change of 17.4 mm (eyes open condition) or 21.4 mm (eyes closed condition) is necessary to confirm a modification of weight-bearing asymmetry. For CE_AREA_, where random error showed an heteroscedastic distribution, a minimal change of 121% or 75% of the initial value (when using SRD_P_) or a minimal change 3.68 or 2.16 ×/÷ the initial value (when using SRD_R_) is required to confirm a modification of postural sway in eyes open or eyes closed condition, respectively. The SEM and SRD data for other CoP-based variables are available in the same way in the Table 2, and could be used to interpret modifications in CoP-based variables over time.

Since SEM or SRD are absolute values, we cannot compare their magnitude between the different CoP-based variables, or with percentage expression. To compare the magnitude of the measurement error between the different CoP-based variables, but not for use as a value of measurement error in clinical practice, we calculated the SRD in percentage of the mean value between sessions 1 and 2 (*SRD*_*MP*_ in Table 2). We obtained a SRD in percentage between 32% and 51% for VEL, SD_VEL_, VEL_ML_ and VEL_AP_, around 145% for CoP_ML_, and between 80% (eyes closed condition) and 131% (eyes open condition) for CE_AREA_. Some authors propose different maximum acceptable values for the SRD (expressed in percentage of the mean value between two sessions) [46,47], ranging from values below 10% or 30%, but there is no consensus or commonly accepted threshold. Our findings showed higher values for all CoP-based variables. For VEL, SD_VEL_, VEL_ML_ and VEL_AP_, values of SRD expressed in percentage were below or equal to 50%. So, despite very high values of ICC, we can consider that these CoP measures showed relatively high measurements errors, and should be used with caution. For CoP_ML_ and CE_AREA_, values of SRD expressed in percentage were above 80%, which confirms their poor reliability. So, we can reasonably assume that these two CoP measures should not be used in clinical practice.

#### Expression of measurement error when heteroscedastic distribution of random error

Based on previous suggestions [21,22], we propose to use the expression of SEM and SRD as percentages (i.e. SEM_P_ and SRD_P_) when random error shows a heteroscedastic distribution, as an alternative to logarithmic transformation. Expression as percentage is suited to a heteroscedastic distribution as defined in this paper because there is a proportional relationship between random error and measurement level. Hopkins also defended an approach based on the use of percentage, which is not substantially biased with respect to data logarithmic transformation [22]. When both methods (i.e. use of percentages or logarithmic transformation) become inadequate because of excessive heterogeneity of the sample, it is advisable to divide the sample into more homogeneous subgroups [22]. But, given the difficulty of forming large cohorts, this subdivision is rarely performed in reliability studies in the field of balance assessment.

SEM_P_ and SRD_P_ give a range of error corresponding to a given percentage of the measured value, which is either symmetrically subtracted or added to it. In contrast, SEM_R_ and SRD_R_ give an asymmetric range of error around the measured value because of their properties, which involve multiplying or dividing by the same factor. The range of error will always be lower below a given measured value than the error above this value [21]. Moreover, the log-antilog procedure gives a slightly higher range of error than a percentage approach on native data (Figure 2B). Thus, because it is easier in and more suited to clinical practice, our results suggest the use of SEM_P_ and SRD_P_ as an alternative to logarithmic transformation in case of heteroscedastic distribution of random error.

### Limitations and perspectives

The limitations of the present study were the quite small number of subjects and the heterogeneity of the population, which may increase the measurement error values calculated. The current reliability data may not be generalized to populations other than hemiplegic stroke patients, or to evaluation methods that would differ in terms of positioning of the feet, length of data acquisition, number of acquisitions in each visual condition, or CoP-based variables used. We emphasize the need for caution in the use of these data in a stroke population because data reliability may be affected by the time since stroke, or the many impairments presented by patients (motor disorders, sensory or cognitive) [25]. Further studies examining the reliability in more homogeneous subset of stroke patients, or offering an anthropometric normalization of variables [48,49], might yield even more relevant data. In the future, conducting study on responsiveness (sensitivity to change) of these CoP-based variables could firstly check that the less reliable are least able to highlight a change over time, and secondly clarify their interest compared to clinical data.

## Conclusions

Results from the present study showed that the use of VEL, SD_VEL_, VEL_ML_ and VEL_AP_, should be preferred over CE_AREA_ to assess postural sway following stroke, and that CoP_ML_ does not seem the most appropriate for assessing weight-bearing asymmetry of stroke patient in clinical practice. Future studies should be conducted to assess the validity of others CoP-based variables [49] in stroke patients.

Using the determination of measurement error in the original unit (e.g., SEM or SRD) without underscoring heteroscedasticity is problematic (as illustrated with CE_AREA_ in this study) because measurement error of small values is overestimated and that of the large values is underestimated. In case of heteroscedasticity of random error distribution, we propose to use SEM and SRD expressed in percentage as an alternative at logarithmic transformation of data to facilitate interpretation of CoP measures variations in hemiplegic stroke patients. In all cases, the use of reliability data from the present study as reference in clinical practice requires a cautious use of absolute or relative values of measurement error in function of the CoP-based variables.

From these reliability data, future work should investigate the responsiveness of CoP measures to determine the relevance of detected changes over time. Finally, the procedure we used could be applied in reliability studies concerning other CoP-based variables or other biological variables in the field of physical medicine and rehabilitation, both in a population of hemiplegic stroke patients in other populations, because appropriately taking into account random error distribution improves the relevance of the results.

## Abbreviations

CoP: Centre of pressure; ICC: Intraclass correlation coefficient; SEM: Standard error of measurement in original unit; SEMR: SEM as a ratio; SEMP: SEM as a percentage; SRD: Small real difference in original unit; SRDR: SRD as a ratio; SRDP: SRD as a percentage; VEL: Mean of resultant CoP velocity; SDVEL: Standard deviation of resultant CoP velocity; VELML: Mean velocity of CoP along the mediolateral axis; VELAP: Mean velocity of CoP along the anteroposterior axis; CEAREA: Area of the 90% confidence ellipse enclosing CoP; CoPML: Absolute value of the mean CoP position along the mediolateral axis.

## Competing interests

The authors declare that they have no competing interests.

## Authors’ contributions

Each of the authors has read and concurs with the content in the final manuscript. DG participated in conceiving the study, carried out acquisition of data, performed the data analysis and carried out the drafting of the manuscript. ML participated in the conception of the study, carried out acquisition of data and helped to draft the manuscript. DA participated in data analysis and has been involved in drafting the manuscript and revising it critically. PD and RM helped to draft the manuscript. PM participated in conceiving and coordination of the study, coordinated the data analysis and supervised the manuscript. All authors read and approved the final manuscript.

## Supplementary Material

Additional file 1 **Detailed clinical characteristics for each patient.** The table detailed age, gender, side of hemiparesis, time after stroke, Fugl-Meyer score, PASS score and FIM score for each patient. Click here for file

Additional file 2 **Graphic illustrations of random error distribution for VEL, SD**_**VEL**_**, VEL**_**ML**_** and VEL**_**AP**_**, both in eyes open and eyes closed conditions.** The figures show random error (i.e. individual test-retest differences) plotted against the individual means of the two sessions for VEL (A), SD_VEL_ (B), VEL_ML_ (C) and VEL_AP_ (D), both in eyes open and eyes closed conditions. For all variables, the random error is rather constant whatever the mean, which suggests absence of heteroscedasticity. Click here for file
