# Supplementary material for: Between-day reliability of centre of pressure measures for balance assessment in hemiplegic stroke patients
Source: J Neuroeng Rehabil. 2014 Mar 21;11:39. doi: 10.1186/1743-0003-11-39 (PMC3999988; doi:10.1186/1743-0003-11-39)
Supplement: Additional file 1 — Detailed clinical characteristics for each patient. The table detailed age, gender, side of hemiparesis, time after stroke, Fugl-Meyer score, PASS score and FIM score for each patient. [file 1743-0003-11-39-S1.pdf]

**Detailed clinical characteristics for each patient**

| Patients (n = 20) | Gender (F/M) | Age (yrs) | Side of Hemiparesis | Time after stroke (mo) | Fugl-Meyer (/34) | PASS (/36) | FIM (/126) |
|---|---|---|---|---|---|---|---|
| S1 | M | 58 | L | 3 | 29 | 32 | 116 |
| S2 | M | 46 | R | 3 | 28 | 34 | 114 |
| S3 | M | 50 | L | 3 | 8 | 33 | 92 |
| S4 | M | 58 | L | 2 | 22 | 28 | 99 |
| S5 | F | 50 | L | 2 | 26 | 34 | 102 |
| S6 | M | 52 | R | 16 | 25 | 34 | 110 |
| S7 | M | 27 | R | 7 | 11 | 23 | 67 |
| S8 | M | 51 | L | 2 | 22 | 33 | 94 |
| S9 | M | 48 | L | 2 | 21 | 33 | 102 |
| S10 | F | 25 | L | 15 | 33 | 35 | 115 |
| S11 | M | 55 | R | 8 | 33 | 36 | 107 |
| S12 | F | 66 | L | 1 | 32 | 36 | 116 |
| S13 | F | 49 | L | 3 | 8 | 33 | 114 |
| S14 | F | 79 | R | 1 | 27 | 34 | 123 |
| S15 | M | 55 | R | 36 | 13 | 33 | 100 |
| S16 | M | 19 | R | 31 | 30 | 36 | 126 |
| S17 | M | 48 | L | 22 | 19 | 33 | 115 |
| S18 | F | 29 | R | 10 | 30 | 36 | 118 |
| S19 | M | 52 | L | 2 | 21 | 33 | 112 |
| S20 | M | 76 | R | 37 | 22 | 34 | 106 |
| Mean (SD) | 14M/6 F | 49.7 (15.5) | 9R/11 L | 10.3 (12.1) | 23.0 (7.9) | 33.2 (3.0) | 107.4 (13.2) |

F, female; L, left; M, male; R, right; PASS, Postural Assessment Scale for Stroke Patients; FIM, Functional Independence Measure.
