# Supplementary material for: Between-day reliability of centre of pressure measures for balance assessment in hemiplegic stroke patients
Source: J Neuroeng Rehabil. 2014 Mar 21;11:39. doi: 10.1186/1743-0003-11-39 (PMC3999988; doi:10.1186/1743-0003-11-39)
Supplement: Additional file 2 — Graphic illustrations of random error distribution for VEL, SDVEL, VELML and VELAP, both in eyes open and eyes closed conditions. The figures show random error (i.e. individual test-retest differences) plotted against the individual means of the two sessions for VEL (A), SDVEL (B), VELML (C) and VELAP (D), both in eyes open and eyes closed conditions. For all variables, the random error is rather constant whatever the mean, which suggests absence of heteroscedasticity. [file 1743-0003-11-39-S2.pdf]

Graphic illustrations of random error distribution for VEL (A), SD_{VEL} (B), VEL_{ML} (C) and VEL_{AP} (D), both in eyes open and eyes closed conditions.

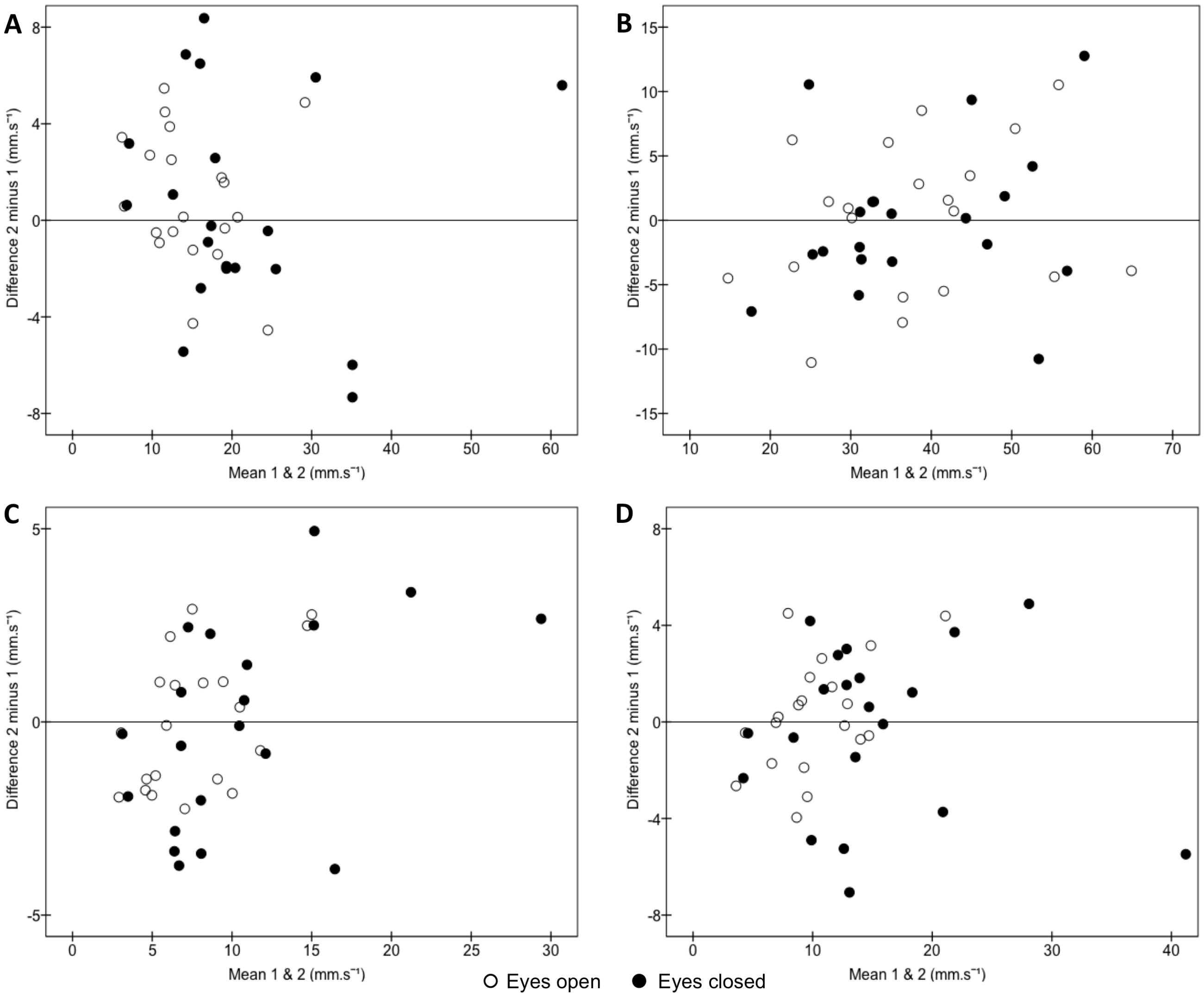

Random error (i.e. individual test-retest differences) is plotted against the individual means of the two sessions. For all variables, the random error is rather constant whatever the mean, which suggests absence of heteroscedasticity.
